# Generosity as a Scientific Method: Building Knowledge and Community in a Competitive World

**DOI:** 10.2196/84933

**Published:** 2026-06-18

**Authors:** Bruno B Andrade

**Affiliations:** 1 Division of Infectious Diseases, Department of Medicine, Johns Hopkins University School of Medicine, 1830 East Monument Street, Room 441, Baltimore, MD, 21287, United States, +1 410 622 6797; 2 Department of International Health, Bloomberg School of Public Health, Johns Hopkins University, Baltimore, MD, United States; 3 Laboratório de Pesquisa Clínica e Translacional, Instituto Gonçalo Moniz, Fundação Oswaldo Cruz, Salvador, Brazil

**Keywords:** generosity, open science, team science, leadership, mentorship

## Abstract

Generosity can function as a scientific method—a disciplined stance that aligns curiosity with openness, credit-sharing, and stewardship of data, specimens, and ideas. Rather than a soft add-on, generosity structures how questions are framed, teams are built, and results are disseminated, thereby improving rigor, reproducibility, and impact. This viewpoint article advances a conceptual and operational framework for “generosity in science,” aimed at researchers, institutions, and funders seeking alternatives to competition-driven models of knowledge production. I examine generosity as practice at the levels of people, collectives, and institutions and argue that persistent global challenges in health demand pro-collaborative architectures. Seen this way, generosity is not mere altruism; it is part of the epistemic engine that turns uncertainty into shared knowledge while distributing opportunity and recognition more fairly. I define core principles of generous research and organize them across three domains: research design, governance, and evaluation. The paper draws on illustrative examples and relevant literature to situate generosity within ongoing debates on open science, team science, and research assessment reform. I outline practical principles for embedding generosity into research design, governance, and evaluation and discuss how these principles can counter vanity metrics and short-term incentives. I conclude that embedding generosity in the infrastructure of science enables better questions, faster learning, and greater public value.

## Introduction: A Different Currency of Success

Science has always been sold to us as a race. A race against time, disease, ignorance, and, above all, against other scientists. The metaphors are warlike: we “fight” cancer, we “combat” pandemics, we “outpace” competitors. This narrative has been useful for funding agencies, for politicians, and for institutions that thrive on the drama of competition. Yet, beneath this spectacle, the real progress of science often emerges from something far quieter and less glamorous: the capacity of human beings to share, to collaborate, and to give.

The book *Infectious Generosity* by Chris Anderson argues that generosity is a kind of contagion, spreading rapidly once individuals and institutions embrace it as a central value [1]. It has made me reflect on how my own scientific journey was shaped less by the victories of rivalry and more by the power of generosity. My most meaningful contributions have been born from collaborations that began with trust and flourished through the act of giving, whether data, time, mentorship, or simply space for others to shine. But having collaborative environments, colleagues, and agencies willing to share (space, reagents, time, and data) is practically a privilege. A growing body of research has demonstrated that reward and recognition systems in science are not neutral, but stratified by geographic, linguistic, gender, and racial criteria, shaping who is cited, funded, and visible in the global knowledge economy [2-6]. In this context, generosity cannot be understood solely as an individual virtue; it is also constrained and enabled by structural conditions that distribute opportunities unevenly. Thus, fostering generosity requires interpersonal practices within laboratories, but also systemic changes in how scientific contribution and value are recognized.

The central question is unavoidable: what happens if we take generosity seriously as a scientific method? If we treat it as a core value of our work, not as a footnote to CVs or a soft skill for committees? This viewpoint article explores how generosity operates in contemporary science, the conflicts it faces with the traditional machinery of academic success, how it may contribute to more inclusive, rigorous, and sustainable forms of knowledge production and why future generations must choose genuine generosity (and not just express it in social media posts) if they are to build a better, more sustainable scientific world.

## Generosity in a Scientist’s Career

When I look back at my career, the turning points did not come from moments when I “outcompeted” someone, even though an outside observer might initially see it that way. In fact, they came from moments when I decided to share, whether it was reagents, ideas, or time. Early on, I realized that mentorship was not a distraction from science but a way to multiply its reach. I invested time in training students, guiding them through complex questions, and giving them the confidence to pursue independent paths. Some of them are now leading their own groups, writing their own grants, and mentoring the next wave. Their success does not diminish mine; it expands it. Each student mentored is like a small wave meeting the shore: gentle on its own, but when joined with others, they gather momentum until they swell into a tide, a force capable of reshaping coastlines. What begins as individual growth becomes a cascading surge of knowledge, leadership, and impact, where today’s student becomes tomorrow’s mentor, carrying the motion forward. In this sense, scientific contribution expands through relational networks rather than remaining confined to individual achievement.

A concrete illustration in my career is the Regional Prospective Observational Research in Tuberculosis (RePORT) International consortium. RePORT links national tuberculosis cohorts—initially in India, Brazil, and South Africa, with expansion to additional sites—through a Common Protocol that standardizes clinical definitions, visit schedules, and the collection, quality assurance, and in-country stewardship of data and biospecimens. Each country builds and governs its own repositories while adhering to shared standards, so that specimens and datasets are interoperable and suitable for pooled analyses. This architecture enables capacity building in high-burden settings, supports South-South and South-North collaborations, and shortens the path from discovery to trial readiness for diagnostics, vaccines, and therapies. By design, RePORT operationalizes generosity: local ownership with shared rules for contribution, access, and credit, thereby creating a repeatable template for translational research networks beyond tuberculosis [7].

Importantly, this model also illustrates how generosity can address structural inequities in global science. By prioritizing in-country stewardship, capacity building, and equitable participation, RePORT challenges traditional models in which data and resources are extracted from high-burden settings and concentrated in institutions in the Global North. Instead, it supports a more distributed model of knowledge production, in which scientific leadership, authorship, and infrastructure development are shared across contexts. In this sense, generosity is not only a collaborative practice, but also a mechanism for redistributing visibility, credit, and opportunity within the global scientific ecosystem.

On a smaller scale, generosity often reveals itself in gestures that leave no trace in metrics or bibliometrics, such as sharing an idea over coffee, connecting a young colleague with a senior researcher, or giving space for others to present first. These acts might not count toward impact factors, but they shape scientific communities in profound ways. Science, at its best, is a collective endeavor, and generosity is the glue that keeps the collective from disintegrating into a pile of isolated egos.

## The Traditional Model of Academic Success

The dominant model of success in academia thrives on scarcity. It measures individuals through h-indexes, citations, and first- or last-authorships, all of which assume that recognition must be a zero-sum game [8]. In this system, your success is interpreted as someone else’s failure, and the currency of prestige is accumulated by exclusion. The system rewards those who guard their data until the moment of publication, who compete for grants as if they were personal trophies, and who see colleagues less as collaborators than as potential rivals.

This model creates an atmosphere of anxiety and mistrust. Young scientists learn quickly that generosity is a liability. Share your idea too soon and someone else might publish it before you. Offer your dataset and you risk losing the first-author paper. Support a colleague’s success too enthusiastically and you might find yourself overshadowed. The traditional model frames generosity as weakness and competition as strength, as if science were a gladiatorial contest rather than a collective search for truth.

There are undeniable achievements produced within this model. Competition drives ambition, and ambition produces results. Yet the costs are increasingly evident. Burnout, toxic lab cultures, plagiarism scandals, and inequities in access to funding all stem from the myth that science thrives when individuals fight each other for crumbs of recognition [9]. The model survives because it is convenient to bureaucracies and profitable to publishers, but it suffocates the very creativity it claims to reward. Actually, this is a dangerous trap: it is impossible to do science alone. And seeing rivalry where there should be collaboration is the key to this individual eventually finding themselves alone—and perhaps here, social and collaborative “failure” carries more weight than the journal cover that was published last month.

## Generosity as Power

Against this backdrop, generosity may appear as an act of resistance, but it is also an act of strategy. Generosity is not weakness—it is power. It transforms influence from something fragile and temporary into something enduring and expansive. A paper might earn your citations, but a culture of generosity creates networks of trust that outlast any citation count.

History provides striking examples. When Jonas Salk developed the polio vaccine, he refused to patent it, famously asking, “Could you patent the sun?” [10]. That act of generosity ensured rapid global access and saved millions of lives. Similarly, the Human Genome Project embraced an ethos of open data sharing through the Bermuda Principles, requiring that sequence data be released within 24 hours [11]. This act of generosity neutralized private efforts to monopolize the human genome and revolutionized biomedical research. In addition, this approach operationalized generosity through data governance, enabling global participation and limiting the consolidation of knowledge within proprietary systems.

More recently, the COVID-19 pandemic showed how generosity scales. The unprecedented speed of vaccine development was made possible by rapid, open sharing of viral sequences [12]. The alternative—secrecy, proprietary data, nationalistic hoarding—would have prolonged the catastrophe. Generosity saved lives, while selfishness would have cost them. These examples suggest that, under certain conditions, openness and shared access can function as accelerators of discovery, particularly in contexts requiring coordinated, transnational action.

The examples discussed throughout this viewpoint are illustrative and were selected to clarify how generosity can operate across different scales of scientific practice. They are intended to support conceptual development and practical reflection rather than to constitute a systematic or scoping review of the literature. Across these cases, generosity operates through identifiable mechanisms: open access to data, equitable distribution of credit, and collaborative infrastructure that lowers barriers to participation. These mechanisms transform generosity from an individual disposition into a reproducible feature of scientific systems.

In my own career, I have seen how generosity amplifies influence across continents. When you mentor generously, your students carry your ethos into their own careers, spreading it in places you will never visit. When you share credit generously, collaborators are eager to return with new ideas and resources. When you share knowledge generously, your field grows stronger and your own work finds deeper resonance. Generosity multiplies success, while selfishness isolates it. Furthermore, when mentees are not recognized for their contributions, they may grow disenchanted with science not because of its true potential, but because of failures in mentorship. This neglect can dull the spark of curiosity and drive, pushing away brilliant minds who might have flourished if nurtured. In the end, selfishness not only isolates success; it risks silencing futures that could have expanded the field itself. The “takers” of science may enjoy brief moments of visibility, but their legacies are fragile, sustained only as long as they can monopolize attention.

## The Future of Science Through Generosity

Future generations face problems so vast that the old model of academic rivalry will collapse under their weight. Climate change, global pandemics, and the inequities of health cannot be solved by isolated laboratories fighting for primacy. These challenges demand collaborations that cross borders, disciplines, and generations [13]. For such collaborations to thrive, generosity must become the rule, not the exception.

Teaching young scientists to embrace generosity is not sentimentalism, it is survival. Rather than framing success solely in terms of individual achievement, mentorship and education can emphasize collaborative capacity, responsible data sharing, and equitable participation in knowledge production. In this context, generosity is not a form of self-sacrifice, but an investment in collective resilience—and generosity should extend beyond academic walls, whether in health care services, efforts to reduce economic and access inequalities, or general well-being [14-16].

When scientific activity is pursued primarily for individual recognition, concerns about priority and visibility may constrain collaboration and slow the circulation of knowledge. In contrast, when the purpose is to nourish science, empowering others is not a threat but a natural extension of one’s mission. By expanding capacity and enabling new voices, the mentor amplifies the very contribution they set out to make, ensuring it reverberates far beyond individual achievements.

Institutions, too, must change. If universities and funding agencies continue to reward individual accumulation above collective impact, they will train generations of scientists ill-prepared for the realities ahead. Instead, we need metrics that value team science [17], platforms that encourage data sharing, and recognition systems that highlight mentorship and collaboration. Generosity must be made visible, measurable, and desirable.

In addition, emerging forms of digital publishing and open science infrastructures create new opportunities to make generosity more visible and traceable. Expanded reference lists, open peer review, data citation practices, and alternative metrics allow for broader recognition of intellectual contributions that extend beyond traditional authorship. These developments suggest that, rather than remaining invisible, many forms of generous scientific practice can be formally acknowledged and integrated into evaluation systems, supporting a more inclusive and representative scholarly ecosystem.

## Framework for Incorporating Generosity Into Science

### Domains and Principles

To translate generosity from a utopian value into a reproducible characteristic of scientific practice, this article proposes a framework organized into three domains: research planning, governance, and evaluation. Within each domain, generosity should be operationalized through fundamental principles, associated practices, and metrics that can help guide implementation (readjusting whenever necessary). Across all domains and areas of knowledge, four interrelated principles underpin this framework: (1) open science (facilitating the timely and responsible sharing of data, methods, and knowledge), (2) equitable recognition (ensuring fair recognition of contributions across all roles, career stages, and geographic regions), (3) collaborative planning (structuring research questions and processes to allow for meaningful participation across disciplines, expertise, and contexts), and (4) management (maintaining responsible, long-term care of data, resources, and scientific relationships).

Scientific participation and recognition are shaped by structural inequities related to geography, language, institutional resources, gender, and race. As a result, the implementation of generosity must explicitly account for these asymmetries. Without such consideration, practices intended to promote openness and collaboration may inadvertently reproduce existing disparities rather than mitigate them.

### Operational Domains

#### Research Design

Generosity in research design involves incorporating external collaboration and accessibility into the study. This includes integrating data-sharing plans into study protocols, identifying potential multisite or interdisciplinary collaborating groups, and anticipating the later reuse of data, samples, and materials. Indicative metrics may include a minimum number of prespecified data-sharing plans, the number of collaborating institutions, and the reuse of datasets in secondary analyses.

#### Governance

Governance structures determine how resources, decisions, and recognition are distributed within scientific collaborations. In this domain, generosity also requires actively paying attention to global and structural inequities, including ensuring that researchers from historically underrepresented regions and groups are not limited to roles of data collection but are meaningfully included in leadership, authorship, and decision-making processes.

Generous governance includes transparent authorization and contribution criteria; shared decision-making processes; and equitable access to data and materials. Support for capacity building (infrastructure, resources, analysis, computing power, among others), particularly in resource-limited contexts. Indicative metrics may include the inclusion of early-career researchers in leadership roles and documented policies for data access and recognition attribution.

#### Evaluation

Evaluation systems shape which behaviors are rewarded in science. Incorporating generosity into evaluation requires recognition of collaborative outcomes (eg, shared datasets, consortium work) and clear inclusion of team contributions to the work, funding, and awards received by the group, among others. Indicative metrics may include citations of shared datasets; participation in multicenter studies; documented contributions beyond first/last authorship; number of students at different levels of knowledge present in each article; and metrics of representation of gender, race/ethnicity, and areas of academic training.

To operationalize generosity, institutions and research groups can adopt practical measures such as (1) incorporating data-sharing plans and timelines into study designs; (2) establishing transparent authorship and credit allocation criteria; (3) recognizing mentorship and collaborative contributions in promotion and funding decisions; and (4) developing indicators that capture collaborative outputs, such as shared datasets, multisite studies, and cross-disciplinary publications.

### Integration

**Figure 1.**
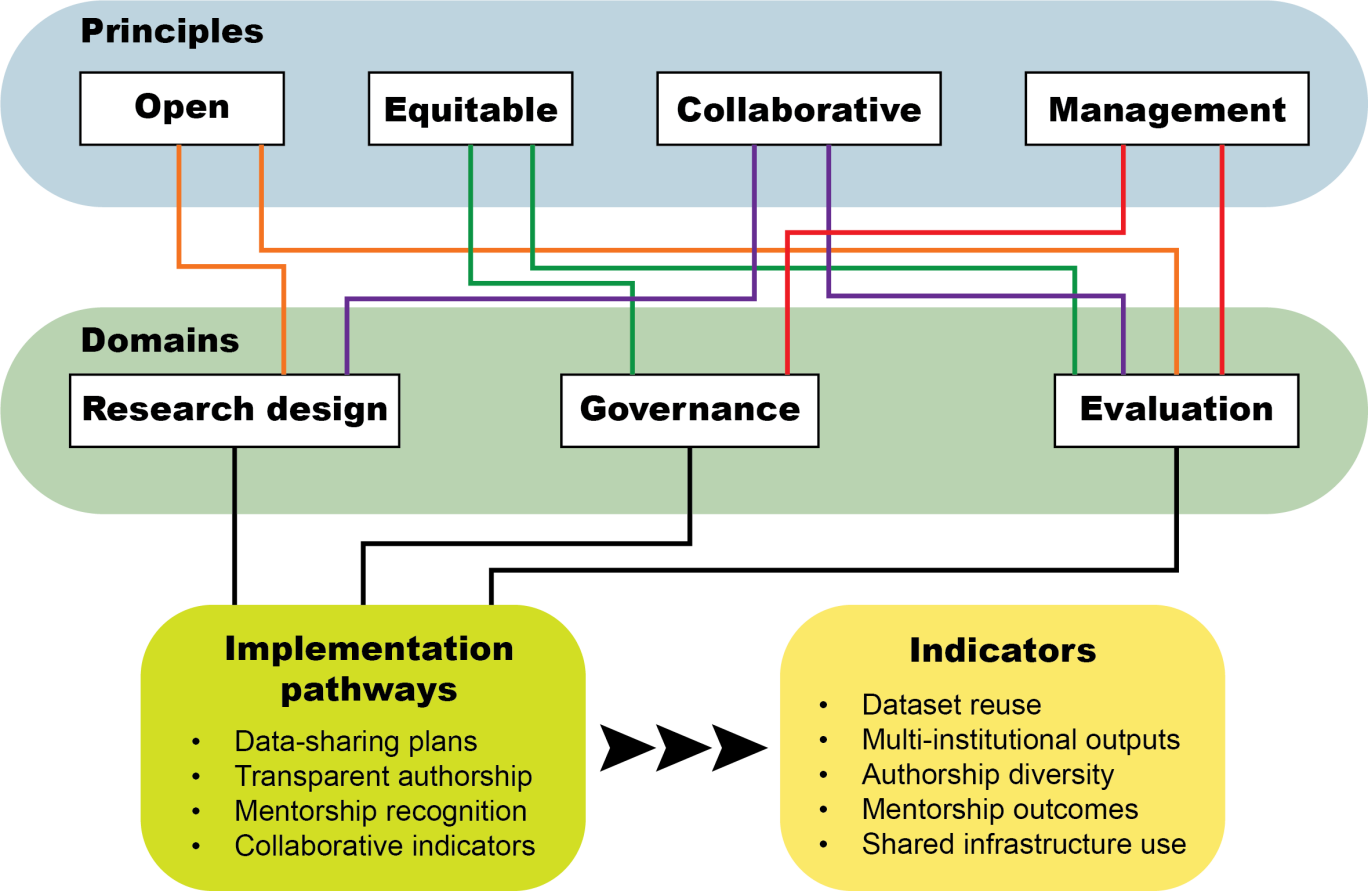
Operational framework for embedding generosity in science. This figure illustrates a multilevel framework for embedding generosity in scientific practice. Core principles (top) inform three operational domains (middle), which are translated into actionable implementation pathways (bottom). Indicators provide mechanisms for evaluation and integration into research systems.

Together, these elements position generosity not as an abstract ideal, but as an actionable and evaluable dimension of scientific practice (Figure 1). By integrating principles, domains, and indicators, this framework provides a structured approach for aligning collaborative values with the operational realities of research systems. Importantly, it allows generosity to be recognized, implemented, and assessed across different contexts, from individual projects to large-scale institutional initiatives. In doing so, it lays the groundwork for a shift from competition-centered models toward more collaborative, equitable, and sustainable forms of knowledge production.

### Limitations

The framework proposed here is conceptual and intended to guide reflection and implementation rather than prescribe a universal model. Its adoption may face important constraints, including intellectual property considerations, regulatory requirements, time-sensitive research environments, and institutional evaluation systems that continue to privilege individual productivity over collective contribution. In some contexts, data sharing may need to be balanced against privacy, biosafety, sovereignty, or ethical concerns. Moreover, the meaning and feasibility of generosity may vary across disciplines, funding structures, and national settings. These boundary conditions do not diminish the value of the framework, but they do indicate that its implementation must be context-sensitive and institutionally supported.

## Conclusion: A Call to Infectious Generosity

This viewpoint has argued that generosity can be understood not only as a scientific virtue, but as a methodological orientation that shapes how research is designed, governed, and evaluated. It guides how we design collaborations, how we mentor, how we share, and how we dream. By aligning practices such as data sharing, equitable credit allocation, and collaborative infrastructure with core scientific goals, it resists the scarcity model and replaces it with abundance, not in the sense of infinite resources, but in the sense of infinite possibilities created through trust.

The framework proposed here highlights how generosity operates across multiple levels (from individual mentorship practices to institutional incentive structures) and identifies actionable pathways for its implementation. At the same time, its adoption is not without challenges, particularly in contexts shaped by competition for funding, intellectual property constraints, and entrenched evaluation systems.

Choosing generosity is not easy in a system that still worships rivalry. It requires courage to give credit when it could have been yours, to share data when secrecy seems safer, to open doors when others are closing them. Yet courage is exactly what science demands of us, and generosity is the most courageous act of all, because it asks us to trust that others will carry forward what we began.

The future of science will be written by those who dare to be generous. If generosity becomes infectious, if it spreads through laboratories and institutions, we may finally build a culture that enables science to deliver on its promises. Not a race, not a war, but a collective journey toward understanding and healing. What kind of research ecosystem do we want to leave for the next generation? One built on rivalry, or one built on generosity? The answer will determine not just the success of our careers, but the fate of our species.

## Abbreviations

RePORT: Regional Prospective Observational Research in Tuberculosis
